# Zancolli procedure and nerve repair with sural graft as a treatment for patient with claw hand due to complete rupture of ulnar and median nerve: A case report

**DOI:** 10.1016/j.ijscr.2018.10.072

**Published:** 2018-11-02

**Authors:** Wahyu Widodo, Agus Waryudi, Zecky Eko Triwahyudi

**Affiliations:** Department of Orthopaedic & Traumatology, Cipto Mangunkusumo National Central Hospital/Faculty of Medicine, Universitas Indonesia, Indonesia

**Keywords:** Median and ulnar nerve, Claw hand, Sural graft, Zancolli procedure

## Abstract

**Introduction:**

Combined injuries of peripheral nerve of upper extremity are usually the result of severe trauma to the extremity, and are often associated with substantial soft tissue, vascular, and bony injuries. The most common form of such combined injury is a low median-ulnar palsy, usually due to laceration of the volar wrist. It is a devastating injury, and with delayed presentation the injury is going to be more difficult to reconstruct.

**Presentation of case:**

Ten-year-old girl was admitted to hospital with numbness of her left palm and fingers, 5 months before admission. She was hit by a car while riding a bicycle from opposite direction from she was heading and cut her left forearm by licensed plate. Initially treated at nearby clinic but later couldn’t extend her fingers after the pain subsided and left untreated for 5 months.

**Discussion:**

Traditionally, number of static procedures have been described to correct the claw hand deformity by preventing MCP joint hyperextension. Nevertheless, these procedures are reserved for those having ability to extend IP joints while MCP joint hyperextension were prevented. Conversely, static procedure like Zancolli capsulodesis is simple and do not sacrifice any motors in an already compromised extremity. Combined with sural graft, the outcome for the patient is hopefully better than Zancolli procedure alone.

**Conclusion:**

Zancolli procedure along with nerve repair with sural graft as a treatment of claw hand due to complete rupture of ulnar and median nerve is one of feasible treatment options to correct claw hand deformity with such combined palsy.

## Highlights

• This case is very devastating due to loss of hand function to the extreme level due to combined injury of ulnar and median nerve.
• There have been very few documentations about this type of injury in English literatures.
• This combination technique of surgery provide excellent outcome for the patient.

## 1 Introduction

Either ulnar nerve palsy or median nerve palsy is considered a more devastating injury than radial nerve palsy [1]. In nerve palsy, key pinch is lost because of absent adductor pollicis and first dorsal interosseous muscle function. Clawing occurs because of paralysis of the interosseous muscles in the presence of functioning extrinsic finger flexors, and this abnormality alone deems special attention. On the other hand, median nerve palsy is perhaps the most devastating single nerve injury of the upper extremity. Not only is there a loss of fine motor control and opposition, but sensibility is lost over the area of the hand used for precision movements and prehensile functioning. [2].

Combined peripheral nerve injuries are usually the result of severe trauma to the extremity,and are often associated with substantial soft tissue, vascular, and bony injuries. Multiple motor-tendon units may be lacerated and require repair, making them unsuitable donors for tendon transfer. Loss of sensibility and proprioception is often more profound than with single nerve palsies, making reconstruction much more complicated [3]. Outcomes are worse than with single nerve palsies, both because of the lack of donor for tendon transfer and the severity of the associated injuries.

The most common combined injury is a low median-ulnar palsy, usually due to laceration of the volar wrist. It is a devastating injury whose treatment requires restoration of opposition and key pinch, reintegration of metacarpophalangeal and interphalangeal joint flexion, and management of clawing [2]. Moreover, with delayed presentation the injury is going more difficult to reconstruct.

Thereby we presented a case of a ten-year-old girl with clawing and numbness of the palmar and fingers of the left hand that was associated with an injury 5 months before admission. It turned out that she had a complete rupture of ulnar and median nerve of the left hand. This case report has been reported in line with the most recent criteria for case report: SCARE criteria [4].

## 2 Case illustration

A ten-year-old girl was admitted to our general hospital with numbness of her left palm and fingers in the last 5 months before admission. At that time, she was hit by a car while she was riding a bicycle. The car was coming from opposite side, and she fell with her left forearm was sliced by licensed plate of the car. There was a semicircular open wound with active bleeding on the left forearm, and she was in pain. She was brought to a nearby clinic and had her left forearm sutured. After the pain subsided, she felt numbness of her left hand and fingers. In addition, she could not extend her fingers. Finally, the patient decided to seek medical attention and get further treatment at our general hospital.

**Fig. 1:**
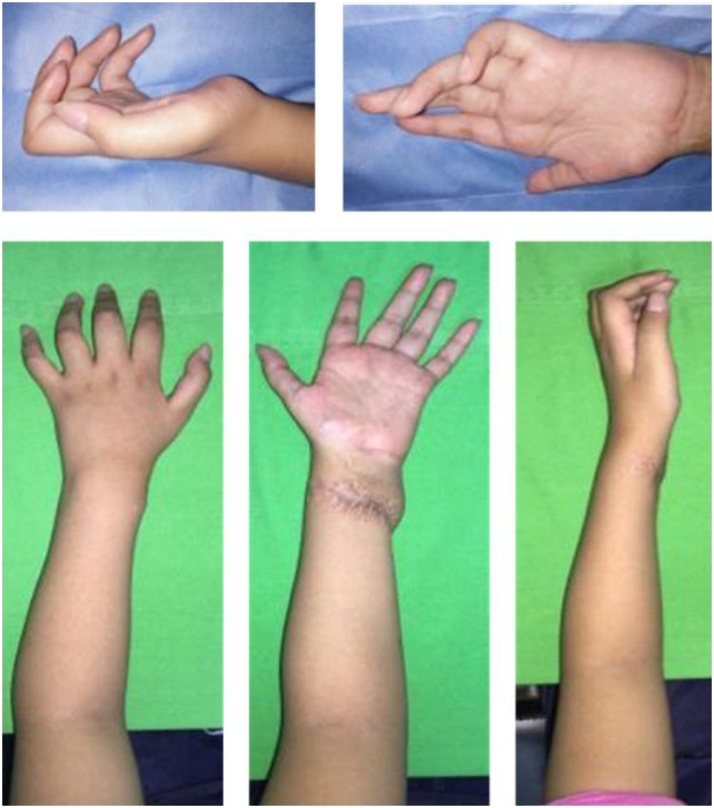
Physical examination showed claw hand deformity with thenar and hypothenar atrophy as well as a scar on the anterior side of distal forearm.

**Fig. 2:**
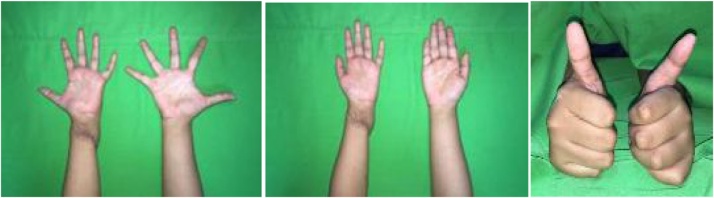
Hand examination revealed limitation of finger abduction and thumb apposition.

From physical examination, there were claw hand deformity with thenar and hypothenar atrophy as well as a scar on the anterior side of distal forearm (Fig. 1). Sensorium loss of the palm and third, fourth, and fifth fingers was impaired. No tenderness was found. Capillary refill of the fingers was normal. Range of motion of the fingers was altered with limitation of finger abduction and thumb apposition (Fig. 2). Moreover, range of motion of the wrist was within normal limit.

Routine laboratory examination was within normal limit. The patient was taken for wrist and forearm radiographs and, similarly, there was no abnormality depicted on either bones or soft tissue.

The patient also underwent electromyography examination which showed median and ulnar nerve lesion at the left forearm with total axonal degeneration. No signs of reinnervation of both peripheral nerves were detected.

The patient was diagnosed as ulnar and median nerve palsy of left forearm, and then we planned to perform surgical exploration of the nerves and to repair with sural nerve graft, Zancolli procedure and sural nerve graft.

**Fig. 3:**
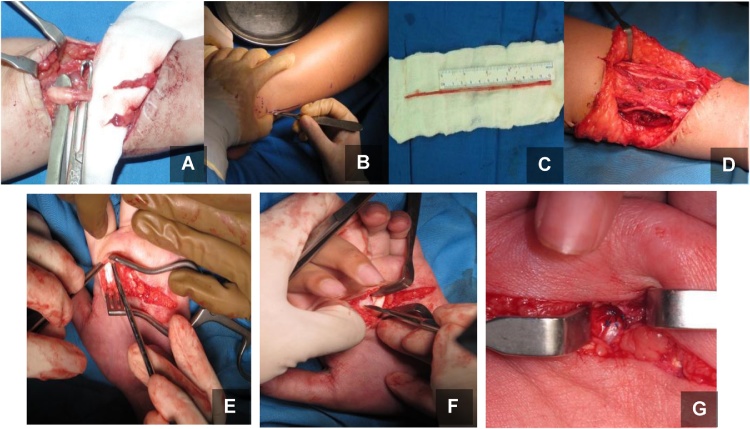
Reconstruction of the hand consisting ulnar and median nerve repair with sural nerve graft and Zancollicapsulodesis procedure.

Intraoperatively, skin incision was made on the previous surgical scar. Injury site was explored, and complete rupture of both ulnar and median nerves was found. Degeneration of both nerves was also seen, with neuroma rising from both the proximal stumps. The proximal and distal ends of both ulnar and median nerves was cut until nerve fascicle was visible. The distance between proximal and distal stump was measured: for ulnar nerve the distance was 7 cm, while it was 8 cm for median nerve. Sixteen centimeters of ipsilateral sural nerve was harvested, and the ulnar and median nerves were repaired using the nerve graft. Then Zancolli procedure was performed: skin incision was made along the palmar crease, A1 pulley was identified around metacarpophalangeal joint, longitudinal incision was made on the pulley, flexor digitorum superficial tendon was retracted laterally, metacarpophalangeal joint capsule was identified, an elliptical incision was made over the joint capsule, and capsulodesis was performed. Postoperatively the wound was closed and immobilized by elastic bandage (Fig. 3).

**Fig. 4:**
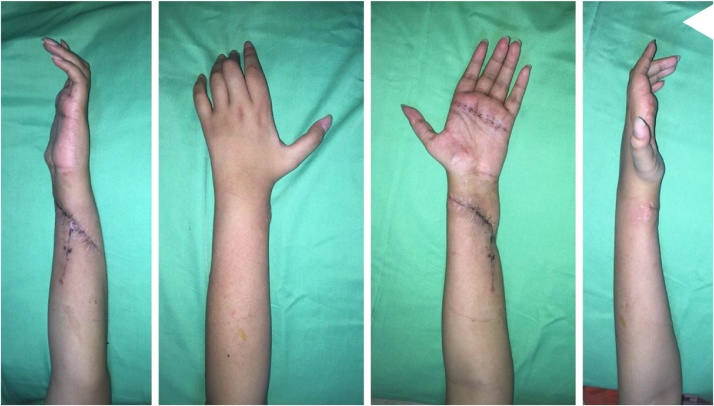
Hand examination of 3-week follow-up revealed improved clawing of the left hand.

**Fig. 5:**
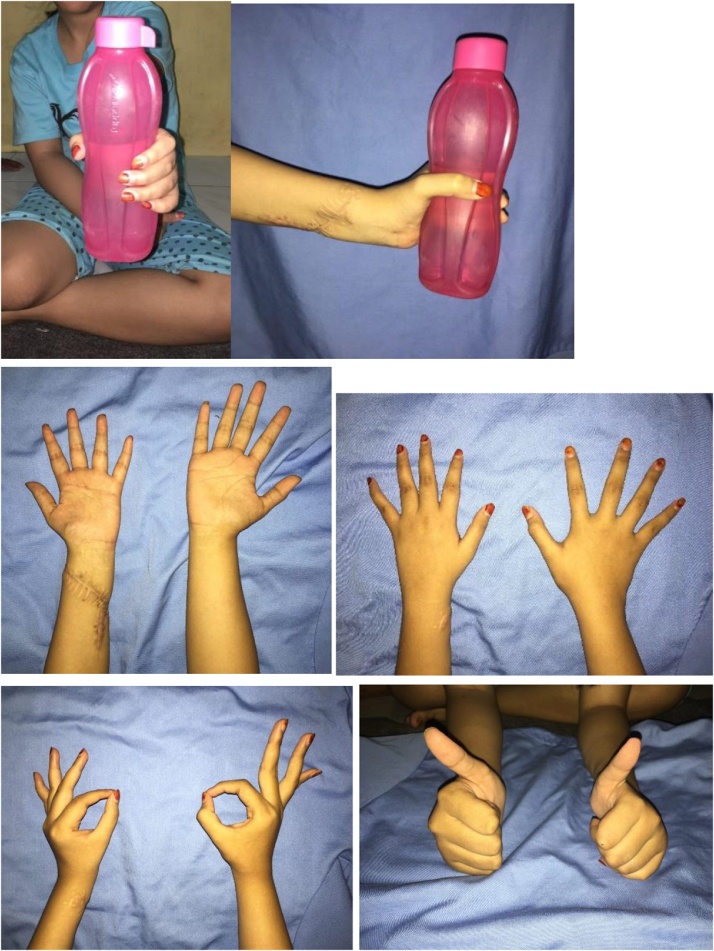
Hand examination at 6-month follow-up.

We followed the patient at 3-week postoperatively, and the patient had improvement of her claw hand (Fig. 4). She was advised to continue her rehabilitation of her hand to further improve her hand function, especially opposition and key pinch. At 6-month follow-up, she had improved grip strength and normal functional level of her left hand. At 2-year follow-up, she could handle daily activity as before the accident and was satisfactory with her condition. (Fig. 5)

## 3 Discussion

The mechanism of injury of our patient was laceration of the volar wrist resulting in combined of low median-ulnar palsy. The treatment for the nerve palsy was delayed for 5 months because the signs of neurological deficit seemed to be unnoticed at that first time she seeks medical attention, making it a late presentation when we met the patient. Physical examination showed obvious signs of neurological deficit: claw hand deformity, thenar and hypothenar atrophy, decreased sensorium of the palm and third, fourth, and fifth fingers as well as limitation of finger abduction and thumb apposition, which consistent with both ulnar and median nerve palsies. This was confirmed by electromyography examination which revealed median and ulnar nerve lesion at the left forearm with total axonal degeneration, without signs of reinnervation of both nerves. Then we decided to perform nerve exploration. Intraoperatively, the diagnosis was established: complete rupture of ulnar and median nerve. In addition, the proximal stumps of both peripheral nerves underwent neuroma formation. From there, we decided to perform reconstruction with Zancolli procedure and nerve repair with sural nerve graft.

Several static procedures have been described to correct the claw deformity by preventing metacarpophalangeal hyperextension. These procedures are reserved for those patients who demonstrate an ability to extend across the interphalangeal joints when metacarpophalangeal hyperextension is prevented [5]. The patient in this case report was easily included in the selection for these static procedures. For patients who are eligible, static procedures act as an internal splint.

The basics of correcting claw hand deformity is to correct the motor imbalance. In patients with ulnar nerve palsy, there is motor imbalance due to absent intrinsic muscle function can produce a claw hand deformity [6,7]. Denervation of the interosseous and lumbrical muscles results in a loss of metacarpophalangeal flexion and interphalangeal extension. Thus, the unopposed extrinsic extensors pull the metacarpophalangeal joints into hyperextension while the unopposed extrinsic flexors pull the interphalangeal joints into flexion. When attempting to open the hand, the extrinsic extensors further hyperextend the metacarpophalangeal joints, thereby forfeiting the excursion that produces interphalangeal extension. The metacarpophalangeal hyperextension also increases the viscoelastic tone of the extrinsic flexors, thereby producing further interphalangeal flexion. Thus, the imbalance of unopposed extrinsic flexors and extensors produces the characteristic metacarpophalangeal hyperextension and interphalangeal flexion of the claw hand deformity. The hand cannot be flattened to reach into narrow spaces and the posture makes it difficult to sweep the fingers around large objects [8].

Tse et al. [4] had provided a list of surgical procedures that can be implemented to treat claw hand deformity in ulnar nerve palsy (Table 1).

**Table 1:** Treatment options for claw hand deformity in ulnar nerve palsy.

Static procedures	Dynamic procedures
Fasciodermodesis	Dynamic tenodesis (Fowler)
Capsulodesis (Zancolli)	FDS tendon transfer
Dorsal metacarpophalangeal bone block	• To lateral band insertion (Stiles-Bunnell)
Static tenodesis	• To phalanx (Burkhalter)
• Free palmaris or plantaris (Parkes)	• To A1 pulley (Zancolli lasso)
• Split ECRL and ECU (Riordan)	Transfer of ECRL with 4-tail tendon graft (Brand)
	Transfer of EIP and EDM

In our center, there were several cases of ulnar nerve palsy due to leprosy that underwent FDS tendon transfer to A1 pulley, that is, Zancolli-lasso procedure [9]. In those cases, The FDS tendon is harvested and wrapped distally around the A1 pulley. The tendon is tensioned and secured to itself to provide active metacarpophalangeal flexion [10,11]. The central principle of FDS tendon transfer is to remove one of the deforming extrinsic forces and to use it to replace the missing intrinsic function [5]. However, it is a dynamic reconstruction surgery, and in traumatic cases like our patient, the motor-tendon units tend to be altered. With our cases, we could not guarantee the FDS tendon was enough to hold the responsibility of intrinsic functions after being transferred.

On the other hand, static procedures such as Zancolli capsulodesis are simple and do not sacrifice any motors in an already compromised extremity. Like other static procedures, the disadvantages of Zancolli capsulodesis are that they do not increase grip strength and the soft tissue procedures invariably stretch out with time allowing recurrence of deformities.

Several techniques for capsulodesis have been described. The common principle of this procedure is to shorten the palmar joint capsule or to fix it to the metacarpal head in such a way to limit metacarpophalangeal extension. The technique is that the volar capsule of each of the four fingers is approached through an incision along the distal palmar crease. The palmar fascia is incised, and the flexor tendons retracted. Several methods of capsular shortening have been described:

1 A simple transverse elliptical excision of the joint capsule and careful closure is used to maintain the joint in 10–30 ° of flexion; [11,12]
2 The capsule is fixed proximally to the metacarpal head using bony drill holes to limit extension to 10–30 ° of flexion; [13]
3 A combination of capsular shortening and proximal fixation to bone may be used to limit joint extension. This involves a distally based capsular flap. Advancement may be facilitated by excision of lateral triangular capsular components [14].

In our case, we decided to perform the first option of capsulodesis. The first option was considered very suitable for the patient because it could restore synchronous finger flexion and spares other superficialis tendon. Furthermore, this choice could result in good thumb ROM. Raskolnikov [12] reported that the mean of MCP hyperextension after this procedure performed was 15°, which is tolerable deformity proven by good DASH score.

The advantages of Zancolli capsulodesis procedure are the procedure quite simple, reproducible with one surgeon, can be performed in line with neurorrhaphy and dynamic procedure, and the result of MCP hyperextension can be reduced in good satisfaction without tendon transplantation or tendon transfer. In the other hand, this procedure could not repair sensory loss of the injured nerve [[12], [13], [14], [15]].

This patient had a complete ruptured of ulnar and median nerve with the result that motoric and sensory impairment. Zancolli [15] stated that the capsulodesis procedure he found could be performed in conjunction to neurorrhaphy procedure. Since Zancolli capsulodesis procedures only corrected claw hand the hyperextension of MCP [12,13], a sural nerve graft was needed in this case to repair the injured nerves, especially the sensory function of intrinsic muscle. After 2 years postoperative follow-up, the patient had the sensory function of the injured hand back in functional.

One of the weak points from this report is that the limited time for follow up. In our knowledge, there are no study that follow the growth of the child with corrected claw hand and how these procedures affect his or her growth. If time and resource permit, we’d like to follow up her until she reaches adulthood and monitor the outcomes.

## 4 Conclusion

Zancolli procedure along with nerve repair with sural graft as one of the treatment options for patients with claw hand due to ulnar and median nerve palsy.

## Conflicts of interest

Nothing to declare.

## Funding source

Nothing to declare.

## Ethical approval

This case is case report; appropriate informed consent has been obtained from the parent for publication for this case report and accompanying images. Ethical approval for this case report has been exempted by our ethical committee.

## Consent

Appropriate informed consent has been obtained from the parent of the patient for publication for this case report and accompanying images. A copy of written consent is available for review on request.

## Author contribution

Agus Waryudi, Wahyu Widodo – examining patient, following up patient, writing the manuscript up and reviewing literatures.

Zecky Eko Triwahyudi – helped and revised this manuscript and adding some literatures.

Wahyu Widodo – senior orthopaedic surgeon assigned to this case.

## Registration of research studies

Nothing to declare.

## Guarantor

Wahyu Widodo, M.D. (corresponding author).

Orthopaedic Surgeon, Hand Consultant at Department of Orthopaedic and Traumatology, Faculty of Medicine Universitas Indonesia.

## Provenance and peer review

Not commissioned, externally peer reviewed.
